# Loss of Inhibin Negative Feedback to Pituitary Gonadotropes Leads to Enhanced Ovulation but Pregnancy Failure in Mice

**DOI:** 10.1210/endocr/bqaf142

**Published:** 2025-09-24

**Authors:** Yeu-Farn Lin, Emilie Brûlé, Luisina Ongaro, Xiang Zhou, Yangfan Jin, Hailey Schultz, Mitra Cowan, David G Stouffer, Ali Yaman, Richard J Auchus, Ulrich Boehm, Daniel J Bernard

**Affiliations:** Department of Pharmacology and Therapeutics, McGill University, Montréal, QC H3G 1Y6, Canada; Department of Anatomy and Cell Biology, McGill University, Montréal, QC H3G 1Y6, Canada; Department of Pharmacology and Therapeutics, McGill University, Montréal, QC H3G 1Y6, Canada; Department of Pharmacology and Therapeutics, McGill University, Montréal, QC H3G 1Y6, Canada; Department of Pharmacology and Therapeutics, McGill University, Montréal, QC H3G 1Y6, Canada; Department of Anatomy and Cell Biology, McGill University, Montréal, QC H3G 1Y6, Canada; McGill Integrated Core for Animal Modeling (MICAM), McGill University, Montréal, QC H3A 1A3, Canada; Department of Internal Medicine, University of Michigan, Ann Arbor, MI 48109, USA; Department of Internal Medicine, University of Michigan, Ann Arbor, MI 48109, USA; Department of Internal Medicine, University of Michigan, Ann Arbor, MI 48109, USA; Department of Pharmacology, University of Michigan, Ann Arbor, MI 48109, USA; Department of Experimental Pharmacology, Center for Molecular Signaling, Saarland University School of Medicine, Homburg 66421, Germany; Department of Pharmacology and Therapeutics, McGill University, Montréal, QC H3G 1Y6, Canada; Department of Anatomy and Cell Biology, McGill University, Montréal, QC H3G 1Y6, Canada

**Keywords:** inhibin, FSH, pituitary, folliculogenesis, infertility

## Abstract

Follicle-stimulating hormone (FSH) is an essential regulator of ovarian function. Inhibins are transforming growth factor β (TGFβ) family ligands produced in the gonads that suppress FSH synthesis by pituitary gonadotrope cells. Inhibins require a coreceptor, betaglycan or TGFBR3L, to mediate their actions. Female mice with a gonadotrope-specific knockout (KO) of betaglycan or global deletion of *Tgfbr3l* have increased FSH activity or levels and produce larger litters compared to controls. Females with both coreceptors knocked out (hereafter dKO) have dramatically increased circulating FSH, ovulate about 4 times as many eggs in natural cycles as controls but are infertile. Here, we show that dKO females show an increased number of implanted embryos at 7.5 days post coitum (dpc) but that their pregnancies fail around mid-gestation. Wild-type surrogates give birth to live young following transplantation of embryos from control or dKO females. Conversely, control but not dKO females can carry wild-type embryos to term, suggesting that the maternal environment in dKO mice cannot support full-term pregnancies. Elevated estradiol (E2) levels are deleterious to pregnancy in mice, and we detected increased E2 production in ovaries of pregnant dKOs. Treatment of these animals with aromatase inhibitors or a selective estrogen receptor degrader increased fetal survival. The results indicate that loss of inhibin action in murine gonadotropes results in excess E2 during pregnancy that precludes successful pregnancy.

The production of healthy oocytes and eggs is necessary for successful reproduction. Oocytes reside in ovarian follicles, which mature through both hormone-independent and -dependent mechanisms (1). Most follicles that are recruited to mature from the quiescent primordial pool will die through an apoptotic process called atresia (1). Those that advance to the antral or early antral stage can be selected and stimulated to rapidly grow by follicle-stimulating hormone (FSH) from the pituitary gland (2, 3). FSH-supported follicles may go on to ovulate within days or weeks, depending on the species (4-7). In the context of assisted reproductive technologies, exogenous FSH is used to increase the number of viable follicles and, ultimately, fertilization-competent eggs (8).

FSH production by pituitary gonadotrope cells is stimulated by gonadotropin-releasing hormone (GnRH) from the brain and activin-class ligands of the transforming growth factor β (TGFβ) family from skeletal muscle and perhaps other sources, at least in mice (9). In contrast, FSH synthesis is suppressed by other TGFβ family ligands produced in the gonads, the inhibins (10, 11). Inhibins are functional antagonists of the activin-class proteins (myostatin, growth differentiation factor 11, and the activins) (9, 12-14). Activin-class ligands drive FSH production by binding to complexes of type I and II receptor serine/threonine kinases on gonadotropes (9, 15). Inhibins, on the other hand, bind to type III coreceptors, allowing them to form high-avidity ternary complexes with activin type II receptors (12). By sequestering type II receptors, inhibins attenuate signaling by activin-class ligands, thereby decreasing FSH levels (12-14).

There are two inhibin coreceptors on gonadotropes: betaglycan (or TGFBR3), which binds both inhibin A and B, and TGFBR3L, which is inhibin B-specific (13, 14). These proteins are necessary for inhibins to exert their actions in gonadotropes, as seen in knockout (KO) mice. Gonadotrope-specific betaglycan KO and global *Tgfbr3l* KO females both show increased litter sizes, secondary to increased follicle growth in response to apparently more active FSH, higher FSH levels, or both (13, 14). Inhibin A fails to suppress FSH production in cultured pituitaries from gonadotrope-specific betaglycan KO mice (13). In pituitaries lacking both betaglycan and TGFBR3L, neither inhibin A nor inhibin B can suppress FSH production (14). In vivo, coreceptor double knockout (dKO) females exhibit markedly elevated FSH levels, and increased numbers of antral follicles and eggs ovulated in natural estrous cycles but are nevertheless infertile (14). Female mice producing inhibins with absent or greatly impaired bioactivity similarly show increased FSH levels but are subfertile (16). In neither model were the causes of infertility or subfertility determined.

Here, we investigated the mechanisms underlying infertility in inhibin coreceptor dKOs and identified a role for excess estradiol (E2) during pregnancy.

## Materials and Methods

### Animal Housing

All animal work was conducted in accordance with federal and institutional guidelines and with the approval of the McGill University Animal Care Committee DOW-A (protocols 5204 and 4437). All animals were housed on a 12-hour light:12-hour dark cycle and given access to food and water ad libitum.

*Tgfbr3* ^fx/fx^ (*Tgfbr3*^tm1.1Hlin^; MGI: 6231174), *Tgfbr3l*^−/−^ (*Tgfbr3l*^em2Djb^; MGI: 6460397), and *Gnrhr*^GRIC/+^ (*Gnrhr*^tm1(cre)Uboe^; MGI: 3795249) mice were previously described (13, 14, 17). *Tgfbr3*^fx/fx^;*Tgfbr3l*^+/−^;*Gnrhr*^GRIC/+^ females and *Tgfbr3*^fx/fx^;*Tgfbr3l*^−/−^;*Gnrhr*^+/+^ males were crossed to generate *Tgfbr3*^fx/fx^;*Tgfbr3l*^−/−^;*Gnrhr*^+/+^ (control) and *Tgfbr3*^fx/fx^;*Tgfbr3l*^−/−^;*Gnrhr*^GRIC/+^ (dKO) animals. Mice were genotyped using the primers listed in Supplementary Table S1 (18).

The following wild-type animals were purchased from Charles River Laboratories: C57BL/6 (strain 027), B6C3F1 (strain 031), and CD1-Elite (strain 482).

### Timed Matings

Unless otherwise indicated, 8- to 12-week-old control or dKO females were mated with adult C57BL/6 males. The morning of the vaginal plug was considered 0.5 days post coitum (0.5 dpc) for the females or embryonic day (E) 0.5 for the embryos.

### Organ and Blood Collection

Animals were euthanized following McGill standard operating procedure 301. At 7.5, 9.5, 10.5, 12.5, and 14.5 dpc, blood, pituitaries, ovaries, and uteri were collected from control and dKO females. The number of implantation sites (including resorptions) were counted immediately. At 7.5 and 10.5 dpc, fetoplacental units were dissected from half the uterus, and the myometrium, embryos, and fetal membranes were removed. Pituitaries, ovaries, decidua (7.5 dpc), and placentae (10.5 dpc) were snap-frozen in liquid nitrogen and stored at −80 °C. The remaining half of the uterus was fixed in 10% neutral buffered formalin (NBF, HT501128, MilliporeSigma) overnight at 4 °C with agitation and stored in 70% ethanol at 4 °C. At 9.5 and 12.5 dpc, the entire uterus was fixed in 10% NBF overnight at 4 °C with agitation and stored in 70% ethanol at 4 °C, while pituitaries and ovaries were snap-frozen in liquid nitrogen and stored at −80 °C.

Unless otherwise indicated, blood was collected by cardiac puncture. Whole blood, regardless of collection method, was allowed to coagulate at room temperature for approximately 30 minutes, then centrifuged at 825*g* for 10 minutes at room temperature. Serum was collected and stored at −20 °C until further analysis.

### Implantation Site Assessment

At 5.5 dpc, control and dKO females were intravenously injected (via tail vein) with 100 μL of 1% Evans blue dye (E2129, MilliporeSigma) dissolved in saline. Five minutes after injection, females were euthanized, uteri were dissected, and implantation sites were counted.

### Natural Ovulation and Embryo Culture

At 0.5 dpc, females were euthanized, and cumulus-oocyte complexes were harvested from the ampullae of both oviducts. Cumulus-oocyte complexes and E0.5 zygotes were digested with 0.5 mg/mL hyaluronidase (H3884, MilliporeSigma) for 5 to 10 minutes at room temperature until dissociated. Oocytes and embryos were purified from cumulus cells and cultured in EmbryoMax Advanced KSOM Embryo Medium (MR-101-D, MilliporeSigma) droplets under mineral oil in a 35-mm dish at 37 °C in a 5% CO_2_ incubator for 96 hours to the late blastocyst stage (E4.5). Oocytes and embryos were imaged on a Zeiss AX10 Observer.Z1 inverted microscope equipped with a Zeiss AxioCam MRm camera and Zeiss AxioVision software v4.6 (Zeiss Canada Ltd).

### Embryo Transfer

Eight- to 9-week-old donor females (control, dKO, or wild-type B6C3F1) were mated with C57BL/6 males (for control and dKO females) or B6C3F1 males (for B6C3F1 females). At 1.5 dpc, females were euthanized and 2-cell embryos were flushed from both oviducts and incubated in KSOM medium droplets under mineral oil in a 35-mm dish at 37 °C in a 5% CO_2_ incubator for 1 hour, until surgical transfer.

In parallel, 8- to 9-week-old surrogate females (control, dKO, or CD1-Elite) were mated with vasectomized CD1-Elite males. At 0.5 dpc, females were anesthetized with isoflurane, an incision made at the midline of the mid-dorsum, and the ovary and oviduct on each side pulled out by the surrounding fat pad. Between 9 and 22 (4-11 per side) 2-cell embryos were transferred into the isthmus of each oviduct using a glass capillary. Females were monitored post surgery. All pregnancies were allowed to go to term and litter sizes were recorded the day of birth. Litters were euthanized at postnatal day 10.

### Embryonic Day 10.5 Embryo Characterization

After euthanasia, the 10.5 dpc uteri were incubated in warm phosphate-buffered saline (PBS) for 1 hour. Embryos were collected, dissected, and imaged using a Leica MZ6 stereomicroscope. Morphological defects (eg, developmental delay and growth restriction, laterality defects, and any other anomalies) were evaluated (19).

### Progesterone Treatment

Progesterone (P4) (P8783, MilliporeSigma) was resuspended in sesame oil at 20 mg/mL. Nine- to 12-week-old pregnant females were injected with 2 mg P4 daily from 6.5 dpc to 17.5 dpc. At 18.5 dpc. blood was collected by submandibular puncture immediately prior to euthanasia. Uteri were dissected and the number of implantation sites (including resorptions) were counted immediately. If fetoplacental units were present, embryo survival was determined by movement. Placentae were fixed in 10% NBF overnight at 4 °C with agitation and stored in 70% ethanol at 4 °C. Pituitaries and ovaries were snap-frozen in liquid nitrogen and stored at −80 °C.

### Aromatase Inhibitor Treatments

Anastrozole (A2736, MilliporeSigma) and formestane (F2552, MilliporeSigma) were resuspended in dimethyl sulfoxide at 40 mg/mL and 300 mg/mL, respectively, and then diluted in sesame oil to 3 mg/mL and 15 mg/mL.

Nine- to 12-week-old pregnant females were subcutaneously injected with 0.15 mg anastrozole, 1.5 mg formestane, or sesame oil daily from 6.5 dpc to 11.5 dpc (as per (20)). At 12.5 dpc, blood was collected by submandibular puncture immediately prior to euthanasia. Fetoplacental units were dissected from the uteri and embryo survival was determined by the presence of a beating heart under a dissection microscope. Placentae were either fixed in 10% NBF overnight at 4 °C with agitation and stored in 70% ethanol at 4 °C, or snap-frozen in liquid nitrogen and stored at −80 °C. Pituitaries and ovaries were snap-frozen in liquid nitrogen and stored at −80 °C.

### Fulvestrant and Placebo Implants

Fulvestrant (ICI 182,780) and placebo pellets (1.5 mg, 12-day release) (custom synthesized by Innovative Research of America) were subcutaneously implanted in 4.5 dpc pregnant females (9- to 12-week-old) (as per (21)). Surgery was performed under isoflurane general anesthesia with standard aseptic techniques following McGill standard operating procedure 201. A small incision in the skin was made on the right side of the neck between the animal's ear and shoulder and the implant situated between the skin and muscle. The incision was closed with wound clips. Animals were euthanized on 12.5 dpc. Embryo survival was assessed and tissues collected as described earlier.

### Histochemical Staining

Fixed placentae were dehydrated in a series of graded ethanol baths (80%, 1 × 1 hour; 95%, 1 × 1 hour; 100%, 2 × 1 hour), cleared with Histoclear (NDIHS-200, Diamed) for 2 × 1 hour, and then embedded in paraffin (18-604-991, ThermoFisher Scientific). Sections were cut at a thickness of 7 μm using a Shandon Finesse 325 microtome. Fixed uteri were processed similarly to placentae, except for the Histoclear step (2 × 30 minutes).

For hematoxylin and eosin staining, tissue sections were deparaffinized at 60 °C for 1 hour, cleared with Histoclear (2 × 5 minutes), and rehydrated in graded ethanol baths (100% and 70%, 5 minutes each). Sections were stained with hematoxylin (Gill No.3, GHS332, MilliporeSigma) and eosin (AC611815000, ThermoFisher Scientific), dehydrated in graded ethanol baths (70% and 100%, 5 minutes each), cleared in Histoclear (2 × 5 minutes), and coverslips mounted with Permount (SP15-100, ThermoFisher Scientific).

For immunohistochemistry, tissue sections were deparaffinized, cleared, and rehydrated as described earlier. Antigen retrieval was performed by incubating tissue sections in Tris-EDTA buffer (10 mM Tris, 1 mM EDTA, 0.05% Triton X-100, pH 9.0) at 95 °C for 20 minutes. Sections were quenched for 15 minutes in 1% H_2_O_2_ diluted in PBS with 0.4% Triton X-100, washed in PBS (3 × 5 minutes), incubated in blocking buffer (5% goat serum [053-110, Wisent], 1% bovine serum albumin [10735086001, MilliporeSigma], 0.4% Triton X-100, in PBS) for 1 hour at room temperature, then incubated overnight at 4 °C with an antibody against vimentin (1:1000, D21H3, Cell Signaling Technology; RRID: AB_10695459) or cytokeratin 8 (1:250, TROMA1c, Developmental Studies Hybridoma Bank, University of Iowa; RRID: AB_531826) diluted in blocking buffer. After overnight incubation, sections were washed with PBS (3 × 5 minutes), incubated with a biotinylated goat anti-rabbit secondary (1:600, BA-1000, Vector Laboratories; RRID: AB_2313606) or donkey anti-rat secondary (1:600, 712-066-153, Jackson ImmunoResearch; RRID: AB_2340649) diluted in blocking buffer for 1 hour at room temperature, washed with PBS (3 × 5 minutes), incubated with avidin-biotin complex (PK-6100, Vectastain Elite ABC, Vector Laboratories) diluted 1:400 in PBS with 0.1% Triton X-100 for 1 hour at room temperature, and washed with PBS (3 × 5 minutes). 3,3′-Diaminobenzidine (DAB, 0.67 mg/mL diluted in Tris-buffered saline, D5637, MilliporeSigma) was used to detect the signal. Sections were counterstained with hematoxylin and then dehydrated, cleared, and mounted as described earlier.

Images were acquired with a Zeiss AxioImager M2 Imaging microscope equipped with a Zeiss AxioCam 506 color camera and Zeiss ZenPro software v3.11 (Zeiss Canada Ltd).

### Image Quantification

All quantitation was performed in Fiji (22). The total uterine area and uterine lumen area were calculated by manually tracing the respective areas. The endometrial area was calculated by tracing the stromal-myometrium junction and subtracting the lumen area. The myometrial area was calculated by subtracting the endometrial and lumen areas from the total uterine area.

### Hormone Quantification–Enzyme-Linked Immunosorbent Assay

Serum LH and FSH were measured using in-house sandwich enzyme-linked immunosorbent assays (ELISAs) as previously described (detection ranges, 0.117 to 30 ng/mL and 0.03125 to 0.5 ng/mL, respectively) (23, 24). Antibodies used were FSH ELISA capture antibody (AFP-1760191, National Institute of Diabetes and Digestive and Kidney Diseases and National Hormone and Pituitary Program [NIDDK-NHPP]; RRID: AB_2665512); FSH ELISA detection antibody (AFP-C0972881, NIDDK-NHPP; RRID: AB_2687903); horseradish peroxidase–conjugated anti-rabbit antibody (AP182P, MilliporeSigma; RRID: AB_92591); LH ELISA capture antibody (518B7, Dr Janet Roser, Department of Animal Science, University of California–Davis; RRID: AB_2665514); LH ELISA detection antibody (AFP-240580Rb, NIDDK-NHPP; RRID: AB_2665533); and ISA horseradish peroxidase–conjugated antibody (P0448, Agilent; RRID: AB_2617138).

Serum P4 was measured by commercial ELISA (IB79105, Immuno-Biological Laboratories Inc; detection range, 0.3 to 40 ng/mL; sensitivity, 0.045 ng/mL; RRID: AB_2892151).

To extract E2 from tissues, ovaries were manually homogenized with polypropylene pestles in 300 μL of PBS and then centrifuged at 16 100*g* for 10 minutes at 4 °C. A total of 200 μL of supernatant was combined with 800 μL of methanol, vortexed to mix, incubated at 37 °C for 10 minutes, then centrifuged at 2000*g* for 10 minutes at room temperature. The supernatant was transferred to a clean glass vial and evaporated to dryness overnight on a 37 °C heat block. The extract was resuspended in 100 μL of standard A (blank), and 50 μL of the sample was immediately assayed in a commercial estradiol ELISA (11-ESTHU-E01, ALPCO; detection range, 20-3200 pg/mL; sensitivity, 10 pg/mL; RRID: AB_2756385).

### Mass Spectrometry

Serum estradiol, estrone, testosterone, and androstenedione were measured by liquid chromatography–tandem mass spectrometry. Aliquots (0.05 mL) of serum were mixed with 0.1 mL high-performance liquid chromatography grade water (W5, ThermoFisher Scientific) and deproteinated with 0.2 mL internal standards in acetonitrile with agitation for 3 to 5 seconds. Samples were incubated at room temperature for 15 minutes, centrifuged for 5 minutes at 12 700*g*, and then chilled at −20 °C for 15 to 20 minutes for cold-induced phase separation. The upper liquid phase was transferred on ice from the frozen lower phase to a glass autosampler vial and dried in a centrifugal vacuum without heating. The dried extracts were reconstituted in 0.3 mL of 0.1 M sodium bicarbonate (424270250, ThermoFisher Scientific) (pH 10.5) and heated with 0.1 mL dansyl chloride (Millipore-Sigma, 1 mg/mL in acetone) at 60 °C for 10 minutes. After chilling at −20 °C for 10 minutes, the reaction mixture was loaded on a supported liquid extraction column (Isolute SLE; Biotage). After soaking into the column under gentle pressure and equilibrating for 5 minutes, the column was washed with 1.75 mL methyl-*tert*-butyl ether over 10 minutes, with pressure applied for 5 seconds twice to complete elution. The eluates were dried in a centrifugal vacuum with a chilled rotor, resuspended in 0.1 mL 1:1 (v/v) methanol:water, and loaded into the autosampler. Derivatized samples (5 μL) were injected and resolved with a 2-dimensional chromatography method via a C_4_, 10 × 3-mm column (Hypersil Gold, ThermoFisher Scientific) on an Agilent 1260 binary pump high-performance liquid chromatography system, followed by a Kinetex 150 × 2.1-mm, 2.6-mm particle-size biphenyl column (Phenomenex) on an Agilent 1290 binary pump UPLC system, using gradient elution with 0.2 mmol/L ammonium fluoride in water and methanol (Supplementary Table S2) (18). Mobile phases consisted of 0.2 mmol/L ammonium fluoride in water (phase A) and 0.2 mmol/L ammonium fluoride in methanol (phase B). The column effluent was directed into the source of an Agilent 6490 triple quadrupole tandem mass spectrometer using electrospray ionization in positive ion mode and analyzed using multiple reaction monitoring (Supplementary Table S3) (18). The lower limits of quantification are as follows: E2 (1 pg/mL), estrone (0.5 pg/mL), testosterone (3 pg/mL), and androstenedione (10 pg/mL).

### RNA Extraction and Reverse-Transcription Quantitative Polymerase Chain Reaction

RNA was extracted from tissues using TRIzol Reagent (15596018; Invitrogen) following the manufacturer's protocol. Pituitaries and decidua were homogenized in 500 μL TRIzol using a Polytron PT10-35 homogenizer; placentae were homogenized in 750 μL TRIzol. Total RNA concentration was determined using a Nanodrop spectrophotometer. Total pituitary (200 ng), decidua (500 ng), and placenta (500 ng) RNA were reverse-transcribed using random hexamers (C1181, Promega) and MMLV reverse transcriptase (M1701, Promega) following the manufacturer's protocol.

Quantitative polymerase chain reaction analysis was performed using BlasTaq (G891, Applied Biological Materials Inc) and primers listed in Supplementary Table S1 (18) on a Corbett Rotorgene 600 instrument (Corbett Life Science). Relative messenger RNA (mRNA) levels were determined using the 2^−ΔΔCT^ method. Gene expression was normalized to ribosomal protein L19 (*Rpl19*). All primers were validated for efficiency and specificity.

### Statistical Analyses

Statistical analyses (with the exception of the Brunner-Munzel test) were performed using GraphPad Prism version 10 software. Unpaired *t* tests with Welch's correction or one-way analysis of variance followed by Dunnett's multiple comparisons test were used to assess statistical significance among experimental groups. The Brunner-Munzel test was performed using the brunnermunzel package (v2.0; Ara, 2022) in R Statistical Software (v4.5.0; R Core Team, 2025). α was set at *P* < .05.

## Results

### Inhibin Coreceptor Knockout Females Ovulate Fertilization-Competent Eggs

**Figure 1.**
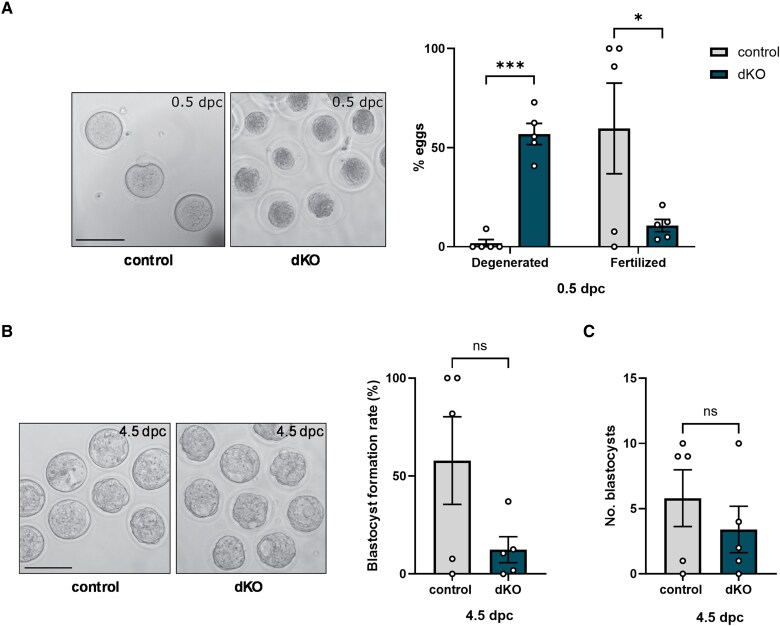
Embryos from double knockout (dKO) mice are developmentally competent in vitro. A, Representative images of eggs/zygotes from control and dKO females at 0.5 days post coitum (0.5 dpc, images at the left) and percentage of eggs that were degenerated or fertilized on that day (quantification at the right). B, Representative images of zygotes from control and dKO females after 4 days in culture (4.5 dpc, images at left) and percentage of eggs that had developed to blastocyst stage on that day (quantification at right). C, Number of blastocysts from control and dKO females on 4.5 dpc. Each dot on the graphs here and in subsequent figures (unless otherwise indicated) represents an individual animal. Results here and in subsequent figures are plotted as mean (bar heights) ± SEM. Data were analyzed by two-tailed unpaired *t* tests with Welch's correction. ns, not significant; *P < .05; ***P < .001.

Female mice lacking functional inhibin coreceptors in gonadotropes (hereafter, dKO) are infertile despite their high FSH levels and the large numbers of eggs they ovulate in natural cycles (14). We therefore asked whether the eggs ovulated by dKO females were fertilization competent. We mated control or dKO females to wild-type males and harvested eggs and zygotes from the oviduct the morning the mating plug was found (0.5 days post coitum, or dpc). Fifty-seven percent of the eggs in dKO females contained degenerated oocytes (characterized by retracted and darkened ooplasm) (25-27), compared to 1.8% in control females (Fig. 1A, Table 1). The percentage of fertilized eggs was also lower in dKOs (16%) than controls (60%) (see Fig. 1A, Table 1). After 4 days in culture (4.5 dpc), only 12% of the eggs/zygotes collected from dKOs had developed to the blastocyst stage, compared to 58% from controls (Fig. 1B, Table 1). However, because dKO females had ovulated more than 4 times as many eggs than controls, the total number of blastocysts per ovulation was comparable between genotypes (Fig. 1C, Table 1). Therefore, dKO produce fertilization-competent eggs that can develop to the blastocyst stage in vitro.

**Table 1.** Fertilization and in vitro developmental competence of mouse embryos

Genotype	No. of females	Total eggs collected	Total fertilized eggs	Fertilized eggs, % total eggs	Degenerated eggs, % total eggs	Blastocyst stage, % total eggs
Control	5	44	30	59.72	1.82	57.90
dKO	5	213	24	15.85	56.85	12.38

Abbreviation: dKO, double knockout.

### Blastocysts Implant Into Uteri of Double Knockout Females

**Figure 2.**
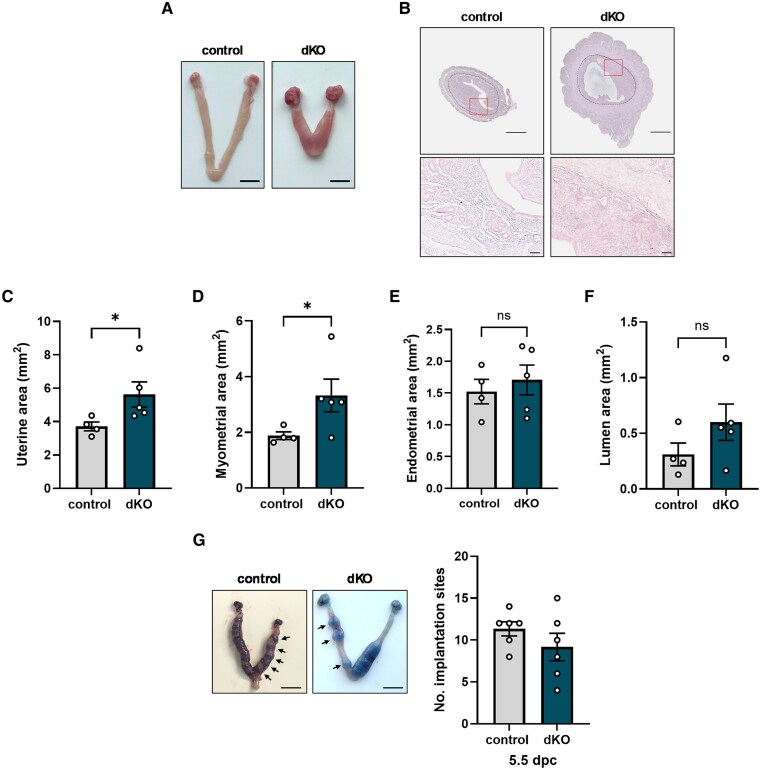
Embryos can implant into double knockout (dKO) uteri. A, Images of control and dKO ovaries and uteri at diestrus. Scale bars: 5 mm. B, Sections of diestrus uteri stained with hematoxylin-eosin. Boxed regions in the top panels are magnified in the bottom panels. Scale bars: 500 μm (top) and 50 µm (bottom). In the top panels, the stromal-myometrium junction is outlined with black dashed lines to delineate the myometrial (outer) and endometrial (inner) areas. The images in A and B are representative of “heavier” uteri seen in most dKO mice. Quantification of C, uterine area; D, myometrial area; E, endometrial area; and F, lumen area during diestrus. G, Representative images of uteri and total number of implantation sites in control and dKO females at 5.5 dpc (left). Scale bars: 5 mm. Arrows indicate implantation sites in one horn of the uterus. Quantification of implantation sites is shown at the right. Data were analyzed by two-tailed unpaired *t* tests with Welch's correction. ns, not significant; *P < .05.

We next asked whether dKOs could produce blastocysts capable of implanting in uteri in vivo. Previously, we noted that the uteri of some dKO mice weighed more than those of controls when assessed at diestrus (14). The heavier dKO uteri appeared shorter and thicker than in controls (Fig. 2A). Changes in the uterus might therefore affect implantation. More granular analyses of uteri sampled on diestrus revealed that total area was greater in dKOs (Fig. 2B and 2C), due to the increased myometrial area (Fig. 2D). The endometrial area was not different between genotypes; the lumina were slightly, but not significantly, enlarged in dKOs (Fig. 2E and 2F). Despite these alterations, we detected similar numbers of implanted embryos both in control and dKO uteri at 5.5 dpc following natural mating (Fig. 2G).

### Pregnancy in Double Knockout Females Fails at Mid-Gestation

**Figure 3.**
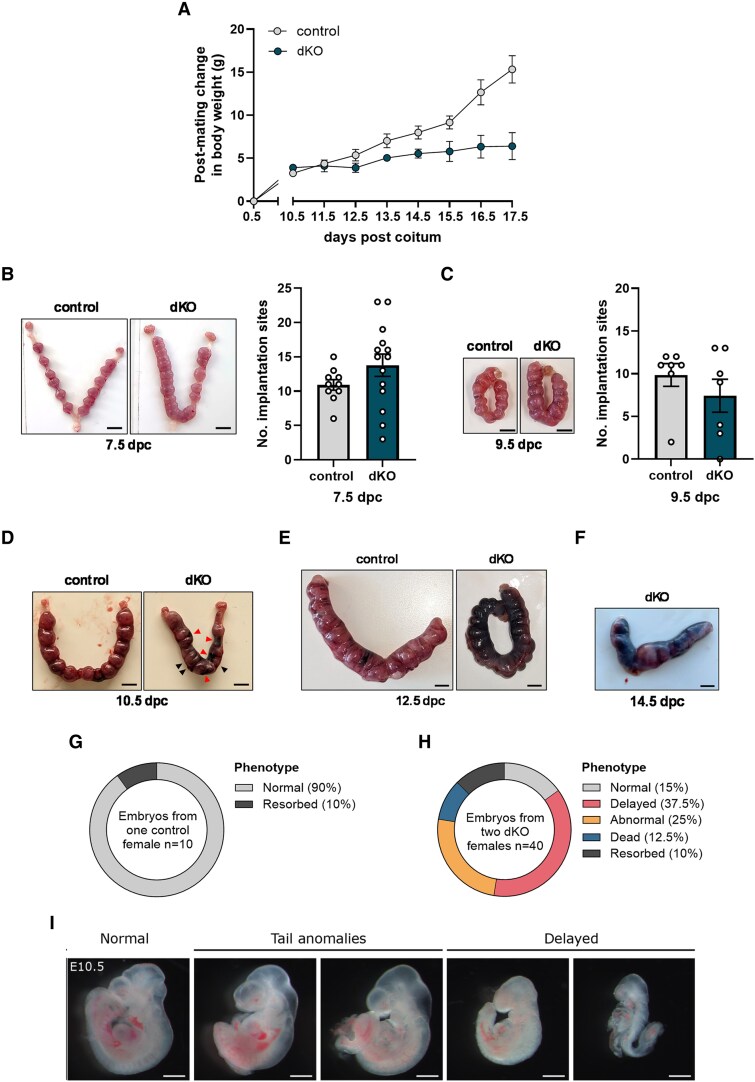
Pregnancy in double knockout (dKO) females begins to fail at mid-gestation. A, Changes in body weight post mating from 0.5 to 17.5 dpc in control and dKO females. N per time point for control females: 8 from 10.5 to 14.5 dpc and 5 from 15.5 to 17.5 dpc. N per time point for dKO females: 11 from 10.5 to 14.5 dpc and 6 from 15.5 to 17.5 dpc. Error bars are SEM. B, Representative images of uteri and total number of implantation sites in pregnant control and dKO females at 7.5 dpc. Scale bars: 5 mm. C, Representative images of uteri and total number of implantation sites in pregnant control and dKO females at 9.5 dpc. Scale bars: 5 mm. Representative images of uteri from pregnant control and dKO females at D, 10.5; E, 12.5; and F, 14.5 dpc (dKO only). Scale bars: 5 mm. In D, black arrowheads indicate smaller fetoplacental units and red arrowheads indicate sites of hemorrhage. Percentages of embryonic day 10.5 (E10.5) embryos that were either morphologically normal, developmentally delayed, morphologically abnormal, dead (blue), or resorbed in G, 1 control litter or H, 2 dKO litters. I, Representative images of E10.5 embryos from dKO litters that were either morphologically normal, had morphological tail anomalies, or had developmental delays. Scale bars: 1 mm.

The data thus far indicated that dKO females produced eggs that could be fertilized in vivo and implant into the uterus. Thus, infertility in these animals results from postimplantation pregnancy failure. To determine when this failure occurs, we weighed the mice throughout pregnancy. Females of both genotypes gained weight after mating, but weight gain in dKOs plateaued around 10.5 dpc. In contrast, control females continued to gain weight until parturition (Fig. 3A). At 7.5 and 9.5 dpc, control and dKO females carried on average the same number of embryos, though the number of fetoplacental units in dKO females was highly variable (Fig. 3B and 3C). Nevertheless, at 7.5 and 9.5 dpc, fetoplacental units in both genotypes looked healthy based on their gross morphology (Fig. 3B and 3C). However, at 10.5 dpc, we began to observe hemorrhagic sites in dKO uteri, and some fetoplacental units in dKOs were noticeably smaller than those in controls (Fig. 3D). At 12.5 dpc, most fetoplacental units in the dKO female were hemorrhagic (Fig. 3E). At 14.5 dpc, almost all fetoplacental units in the dKO female were necrotic (Fig. 3F).

As hemorrhagic sites began to appear in the dKO uteri at 10.5 dpc, we characterized the morphology of embryonic day 10.5 (E10.5) embryos in a litter from 1 control female and litters from 2 dKO females. Ninety percent of the embryos in the control female were morphologically normal (Fig. 3G) compared to only 15% of the embryos in the dKO females (Fig. 3H and 3I). Of the embryos in the dKO females, 37.5% exhibited delayed development, 25% had morphological anomalies, and 22.5% were dead or resorbing (see Fig. 3H and 3I).

### Pregnancy in Double Knockout Females Fails Due to a Maternal Factor

**Figure 4.**
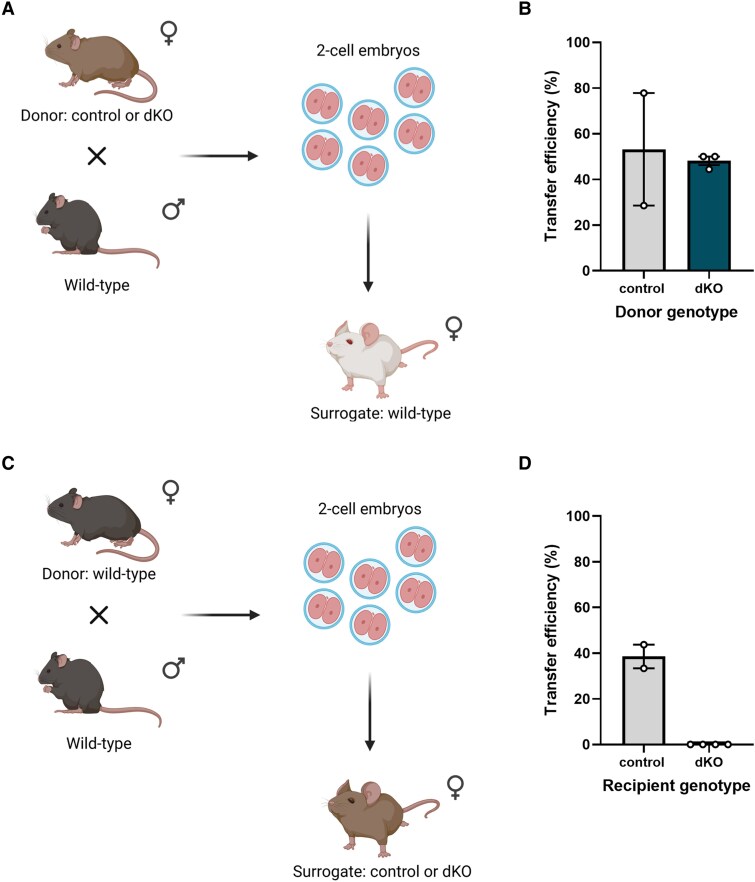
The maternal environment in double knockout (dKO) females cannot support full gestation. A, Schematic of embryo transfer from control or dKO females into wild-type females. B, Efficiency of embryo transfer (percentage of live pups per 2-cell embryo transferred) from control or dKO females into wild-type females. C, Schematic of embryo transfer from wild-type females into control or dKO females. D, Efficiency of embryo transfer from wild-type females into control or dKO females. Due to the low sample size, statistical tests were not performed. A and C, Were created with BioRender.com.

To determine whether pregnancy failure in dKO females was due to a maternal or fetal factor, we performed embryo transfer experiments. We mated control or dKO females to wild-type males, collected 2-cell embryos at 1.5 dpc, and transferred them into pseudopregnant wild-type females (Fig. 4A). These surrogates gave birth to live pups at a similar rate regardless of the genotype of the donor female (Fig. 4B, Table 2). In contrast, when 2-cell embryos from wild-type matings were transferred into pseudopregnant control or dKO females, only control surrogates gave birth to live pups (Fig. 4C and 4D, Table 2). All dKO surrogates gained a minimum of 3.6 g of body weight 2 weeks post transfer (data not shown), suggesting that pregnancy was established but dKO females did not provide a suitable environment for embryo/fetal viability.

**Table 2.** Embryo transfer efficiency

Donor female genotype (No.)	Surrogate female genotype (No.)	No. 2-cell embryos transferred, average	No. live pups, average	Efficiency %, average
Control (3)	Wild-type (CD-1 Elite) (2)	11.5	5.5	53.17
dKO (4)	Wild-type (CD-1 Elite) (3)	28.67	13.67	48.15
Wild-type (B63F1)*^a^*	Control (2)	15.5	6	38.54
Wild-type (B63F1)*^a^*	dKO (3)	16.5	0	0

Abbreviation: dKO, double knockout.

^*a*^The number of wild-type donor females was not recorded.

### Progesterone Deficiency Is not Responsible for Fetal Loss in Double Knockout Females

**Figure 5.**
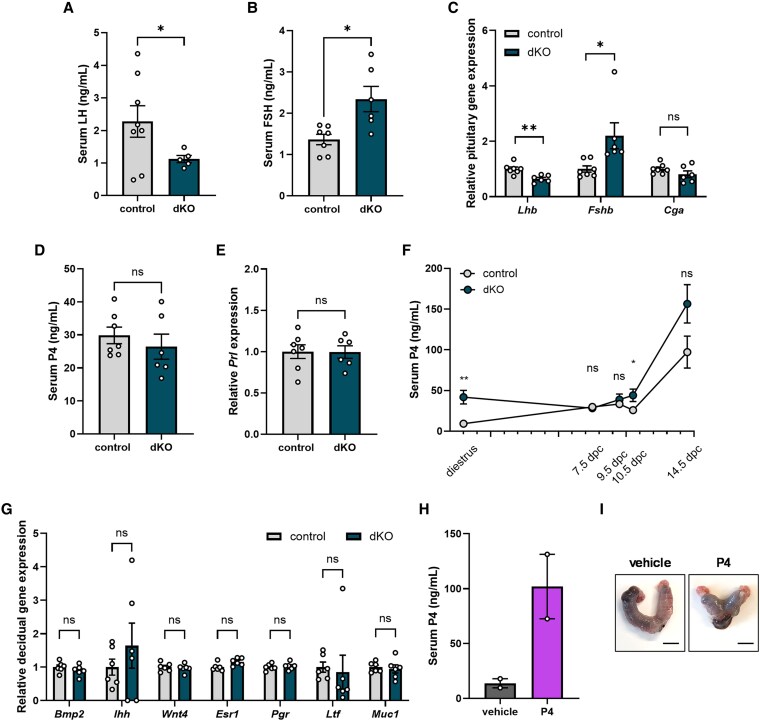
Double knockout (dKO) females produce sufficient progesterone to support pregnancy. A, Serum luteinizing hormone (LH) and B, follicle-stimulating hormone (FSH) in control and dKO females at 7.5 dpc. C, Pituitary expression of *Lhb*, *Fshb*, and *Cga* in control and dKO females at 7.5 dpc. D, Serum progesterone (P4) in control and dKO females at 7.5 dpc. E, Pituitary expression of *Prl* in control and dKO females at 7.5 dpc. F, Serum P4 in control and dKO females at diestrus, 7.5 dpc, 9.5 dpc, 10.5 dpc, and 14.5 dpc. Data from diestrus females were previously published (14) and data from 7.5 dpc animals are the same as in panel D. N per time point for control females: 8 for 9.5 dpc, 11 for 10.5 dpc, and 9 for 14.5 dpc. N per time point for dKO females: 5 for 9.5 dpc, 10 for 10.5 dpc, and 7 for 14.5 dpc. G, Expression of *Bmp2*, *Ihh*, *Wnt4*, *Esr1*, *Pgr*, *Ltf*, and *Muc1* in 7.5 dpc decidua in control and dKO females. Each dot on the graph represents the average expression of 3 decidua from 1 litter. H, Serum P4 in vehicle- and progesterone-treated dKO females at 18.5 dpc. I, Representative images of uteri from vehicle- and progesterone-treated dKO females at 18.5 dpc. Data in A through G were analyzed by two-tailed unpaired *t* tests with Welch's correction; statistical tests were not performed on the data in H due to the low sample size. ns, not significant; *P < .05; **P < .01.

Having established that the postimplantation maternal environment of dKO females was deleterious to embryo survival, we examined the reproductive endocrine profiles of pregnant control and dKO mice. At 7.5 dpc, serum LH and pituitary *Lhb* mRNA levels were decreased in dKO relative to control females, whereas serum FSH and pituitary *Fshb* mRNA levels were elevated (Fig. 5A-5C). There was no genotype difference in pituitary *Cga* mRNA levels (Fig. 5C). Serum P4 at 7.5 dpc was comparable between genotypes (Fig. 5D), despite the greater number of corpora lutea and elevated P4 levels observed previously in cycling dKO females (14). We therefore suspected luteal insufficiency during pregnancy. However, pituitary expression of prolactin, which contributes to maintenance of the corpora lutea during early gestation (28, 29), was comparable between controls and dKOs (Fig. 5E). Also, serum P4, though comparable between genotypes at 9.5 dpc, was slightly elevated in dKO females compared to controls at 10.5 dpc, rose in both genotypes between 10.5 and 14.5 dpc, and was comparable between genotypes at 14.5 dpc (Fig. 5F). We did not observe genotype differences in decidual expression of P4-responsive genes (bone morphogenic protein 2 [*Bmp2*], Indian hedgehog [*Ihh*], and Wnt family member 4 [*Wnt4*]) or E2-responsive genes (estrogen receptor α [*Esr1*], progesterone receptor [*Pgr*], lactotransferrin [*Ltf*], and mucin 1 [*Muc1*]) (30-33) at 7.5 dpc (Fig. 5G).

Nevertheless, to determine whether endogenous P4 levels in dKO females were insufficient to maintain pregnancy, we treated dKO females with 2 mg of P4 daily from 6.5 dpc to 17.5 dpc. This treatment has been effective in treating pregnancy failure due to luteal insufficiency in other mouse models (34). Subcutaneous administration of exogenous P4 increased serum levels at 18.5 dpc but did not rescue pregnancy (Fig. 5H and 5I). Collectively, P4 deficiency seems unlikely to explain pregnancy failure in dKO females.

### Double Knockout Females Have Elevated Ovarian Estradiol

**Figure 6.**
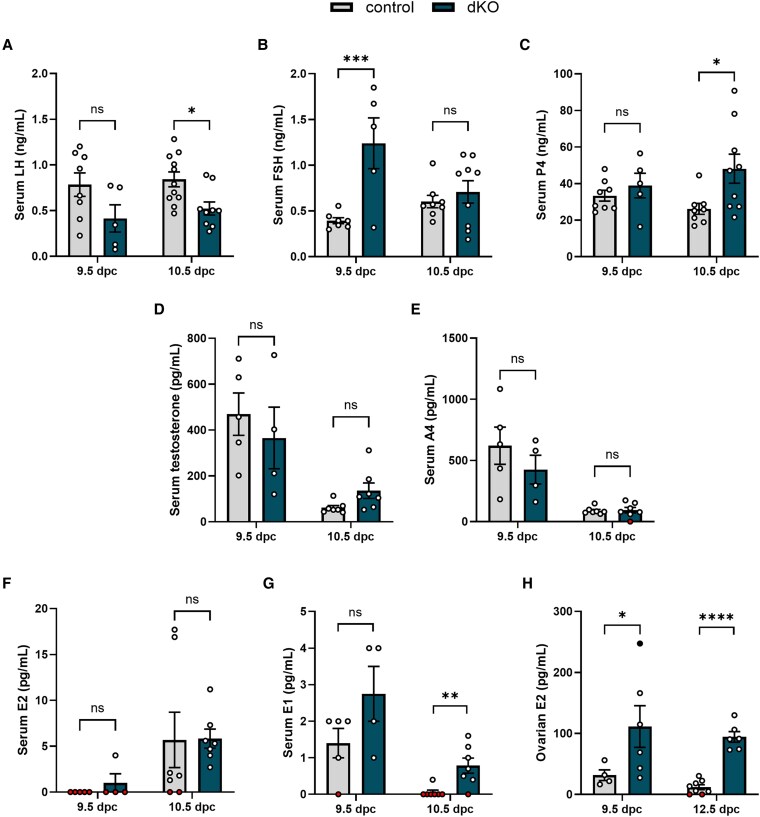
Double knockout (dKO) females produce more ovarian estradiol at mid-pregnancy. Serum A, luteinizing hormone (LH); B, follicle-stimulating hormone (FSH); and C, progesterone (P4) in control and dKO females at 9.5 and 10.5 dpc, measured by enzyme-linked immunosorbent assay (ELISA). Serum D, testosterone; E, androstenedione (A4); F, estradiol (E2); and G, estrone (E1) in control and dKO females at 9.5 and 10.5 dpc, measured by mass spectrometry. H, E2 content of ovaries from control and dKO females at 9.5 and 12.5 dpc, measured by ELISA. Symbols filled in red represent samples in which the hormone was not detectable. The symbol filled in black is referred to in the text. Data in A to E and H were analyzed by two-tailed unpaired *t* tests with Welch's correction. Data in F and G were analyzed by Brunner-Munzel tests. ns, not significant; *P < .05; **P < .01; ***P < .001; ****P < .0001.

The aforementioned analysis focused on early pregnancy. We next examined genotype differences in gonadotropins and steroid hormones at 9.5 and 10.5 dpc, when embryo death starts to occur in dKO pregnancies. Serum LH did not differ significantly between genotypes at 9.5 dpc and was slightly lower in dKOs at 10.5 dpc (Fig. 6A). Serum FSH was elevated in dKOs at 9.5 dpc but did not differ between genotypes at 10.5 dpc (Fig. 6B). Serum P4 did not differ between genotypes at 9.5 dpc but was elevated in dKOs at 10.5 dpc (Figs. 5F and 6C). Serum androstenedione and testosterone did not differ between genotypes at either time point, though the androgens were lower at 10.5 than 9.5 dpc (Fig. 6D and 6E). Serum E2, as measured by mass spectrometry, was mostly undetectable at 9.5 dpc (Fig. 6F). We could measure serum E2 at 10.5 dpc, but levels were low (see Fig. 6F). Serum estrone (E1) could be detected in many samples, but overall levels were also low (Fig. 6G). E1 and E2 were undetectable in a larger proportion of control than dKO animals. At 10.5 dpc, median E1 and E2 were both higher in the dKO animals; however, values were generally near the limit of quantification and not normally distributed. As mice lack sex hormone–binding globulin (35, 36), making accurate E2 measurements in serum difficult (37), we analyzed ovarian E2 content by ELISA. Intraovarian E2 levels were elevated in dKO females compared to controls at 9.5 and 12.5 dpc (Fig. 6H). Interestingly, a female that had almost completely resorbed her fetoplacental units at 9.5 dpc had the highest ovarian E2 content (indicated by the solid black dot). Collectively, the data suggested that E2 was elevated in dKO females around the time of embryo death.

### Decreasing Estrogen Action Rescues Pregnancy in Double Knockout Females

**Figure 7.**
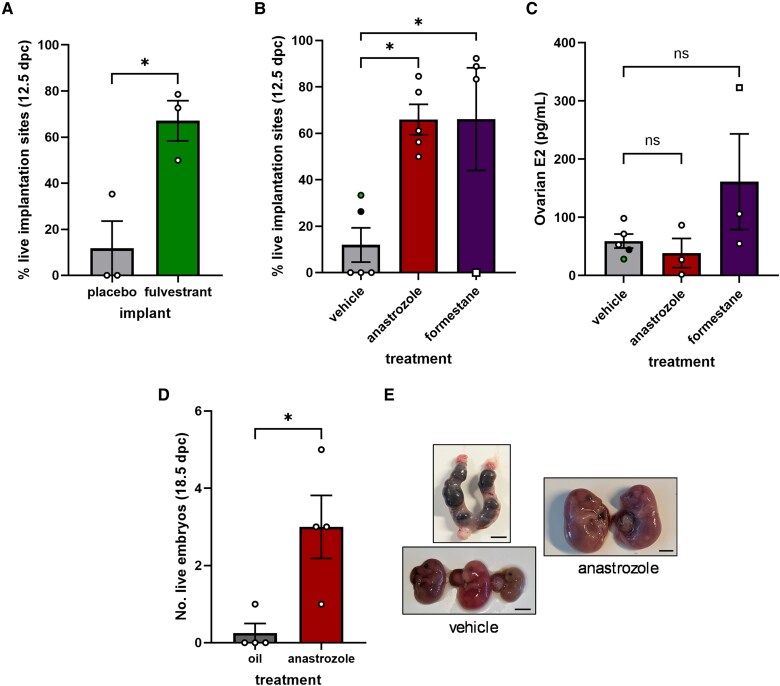
Decreasing estrogen action increased embryo survival in double knockout (dKOs). A, Percentage of implantation sites that contained live embryos at 12.5 dpc in dKO females receiving a placebo or fulvestrant implant. B, Percentage of implantation sites that contained live embryos at 12.5 dpc in dKO females treated with vehicle, anastrozole, or formestane. C, Ovarian estradiol (E2) content of 12.5 dpc dKO females treated with vehicle, anastrozole, or formestane. The green circle, black circle, and white square show data from the same animal in the analyses in B and C, as described in the text. Mismatches in sample sizes between B and C are due to the loss of ovaries during dissections. D, Number of live embryos at 18.5 dpc in dKO females treated with vehicle or anastrozole. E, Representative images of uteri and embryos at 18.5 dpc in dKO females treated with vehicle or anastrozole. Scale bars: 5 mm. Data in A and D were analyzed by two-tailed unpaired *t* tests with Welch's correction. Data in B and C were analyzed by one-way analysis of variance followed by Dunnett's multiple comparisons test. ns, not significant; *P < .05.

Elevated E2 is deleterious to pregnancy in mice (20, 21). To determine whether elevated E2 might cause pregnancy loss in dKO mice, we blocked estrogen action with implants of fulvestrant (ICI 182, 780), a selective estrogen receptor degrader, starting at 4.5 dpc. Embryo survival at 12.5 dpc, as determined by presence of a heartbeat, was significantly increased by fulvestrant treatment (Fig. 7A). Next, we decreased estrogen synthesis by injecting pregnant dKO mice with the aromatase inhibitors anastrozole or formestane, starting at 6.5 dpc. Daily injections of both drugs improved embryo survival at 12.5 dpc (Fig. 7B). Anastrozole slightly, but not significantly, decreased ovarian E2 content, while formestane did not (Fig. 7C). However, it should be noted that intraovarian E2 levels vary markedly in dKO mice (Fig. 6H) and, given the nature of the analysis, we are not able to determine the effect of the drug treatments in individual animals. Interestingly, the 2 vehicle-treated dKO females with the lowest ovarian E2 still had some live embryos at 12.5 dpc (Fig. 7B and 7C, indicated by the solid black and green dots). In contrast, the ovary with the highest E2 content in the formestane condition was from a female in which no embryos were rescued (Fig. 7B and 7C, indicated by a white square).

When extended to 17.5 dpc, anastrozole rescued between 1 and 5 embryos at 18.5 dpc (Fig. 7D and 7E). In contrast, embryos in vehicle-treated dKO females were mostly in the process of resorbing, though we did find 1 undersized live embryo out of the 16 embryos analyzed (see Fig. 7D and 7E). Anastrozole-treated dams sometimes experienced dystocia, as estrogens play a role in cervical ripening (38), but in 2 cases, live births occurred naturally. One anastrozole-treated dKO female produced 10 live pups on 19.5 dpc, and the other produced 4 live pups on 22.5 dpc. Thirteen of the 14 pups (males and females) survived until weaning and appeared to be of normal size. We did not assess them thereafter.

### Dysregulation of Proangiogenic and Antiangiogenic Genes in Placentae From Double Knockout Females

**Figure 8.**
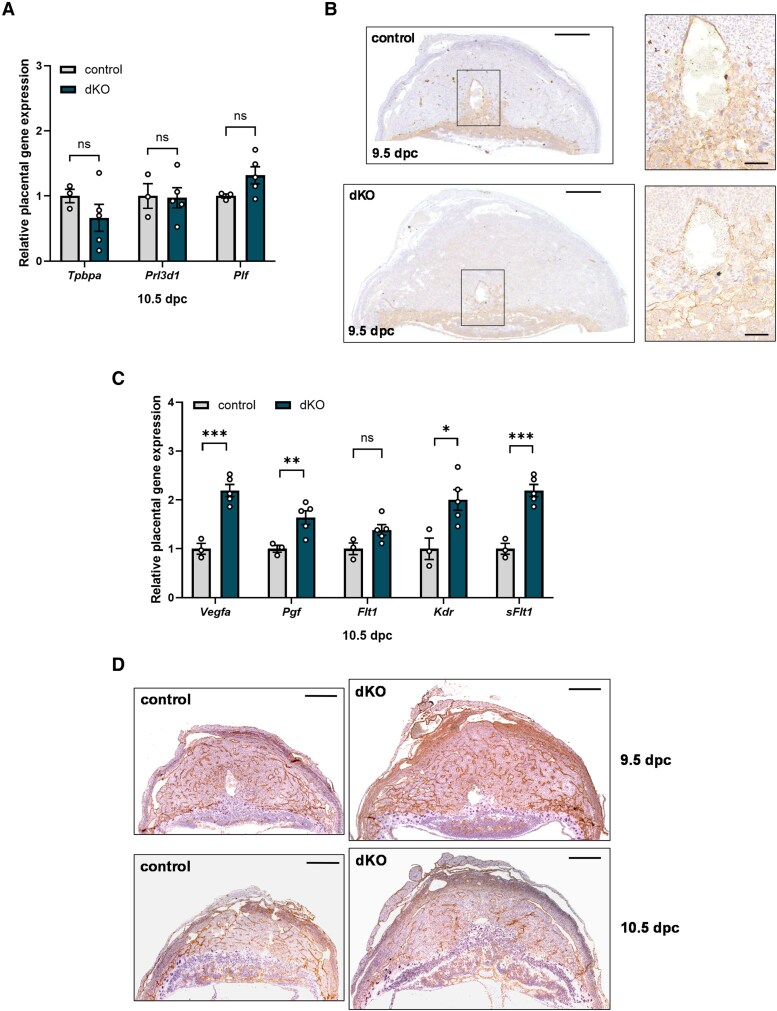
Dysregulated expression of angiogenic genes in placentae from double knockout (dKO) females. A, Placental expression of *Tpbpa*, *Prl3d1*, and *Plf* in control and dKO females at 10.5 dpc. B, Cytokeratin 8 immunostaining in placentae from control and dKO females at 9.5 dpc. Boxed regions (showing the maternal spiral artery) in the left panel are magnified in the right panels. Scale bars: 500 µm (left) and 100 µm (right). C, Placental expression of *Vegfa*, *Pgf*, *Flt1*, *Kdr*, and *sFlt1* in control and dKO females at 10.5 dpc. In A and C, each dot on the graph represents the average expression of 2 to 3 placentae from 1 litter. D, Vimentin immunostaining in placentae from control and dKO females at 9.5 and 10.5 dpc. Scale bars: 500 µm. Data were analyzed by two-tailed unpaired *t* tests with Welch's correction. ns, not significant; *P < .05; **P < .01; ***P < .001.

Angiogenesis and trophoblast-mediated maternal spiral artery remodeling are estrogen-regulated processes vital for proper placentation (39-44). We first investigated trophoblast differentiation and invasion. Here, we randomly selected 2 or 3 placentae from each litter for quantitative polymerase chain reaction analysis and averaged the results from each dam. At 10.5 dpc, we did not observe genotype differences in placental expression of the trophoblast progenitor marker trophoblast specific protein α (*Tpbpa*) (Fig. 8A) (45, 46). Expression of placental lactogen-I α (*Prl3d1*) and proliferin (*Plf*, also known as *Prl2c2*) (45, 46), also did not differ between genotypes (Fig. 8A). We then performed histological analyses of whole implantation sites to assess trophoblast invasion and vascular remodeling. In 9.5 dpc placentae from both control and dKO females, trophoblasts (stained by cytokeratin 8) had infiltrated the maternal spiral arteries and replaced the endothelial cells therein (Fig. 8B).

Next, we investigated placental expression of proangiogenic and antiangiogenic genes at 10.5 dpc. mRNA levels of the proangiogenic ligands, vascular endothelial growth factor A (*Vegfa*) and placental growth factor (*Pgf*), were elevated in dKOs, as was the expression of their receptors, fms-related receptor tyrosine kinase 1 (*Flt1*, also known as *Vegfr1*) and kinase insert domain receptor (*Kdr*, also known as *Vegfr2*) (Fig. 8C). The extracellular region of FLT1 also exists in a soluble form (*sFlt1*), which acts as an antiangiogenic decoy receptor that traps VEGF and PGF (47, 48). Expression of *sFlt1* was also elevated in placentae from dKO females (Fig. 8C).

We then examined 9.5 and 10.5 dpc whole implantation sites for any structural changes in the vascular network. At 10.5 dpc, some of the smaller fetoplacental units were in the process of being resorbed, exhibited extensive tissue necrosis, and were thus excluded from this analysis. Labyrinth development was in progress in placentae from both genotypes, and decidual vascular density in placentae from dKOs appeared comparable to controls at these time points (Fig. 8D).

## Discussion

Female mice with loss of inhibin action in pituitary gonadotropes exhibit increased FSH secretion and ovulation but are infertile (14). Here, we show that many of the ovulated eggs are fertilization competent and can form blastocysts that implant in the uterus and develop as seemingly normal embryos to mid-gestation. Pregnancy then fails, with embryos dying at varying time points between 10.5 and 18.5 dpc. Some embryos can be rescued in dKOs by aromatase inhibitors or an estrogen receptor degrader. These results suggest that supraphysiological levels of E2 contribute to embryo death in this model. Nevertheless, we were unable to directly demonstrate increases in circulating E2 (though ovarian E2 content was increased) or a mechanism underlying E2-dependent embryo loss.

### Is Estradiol Production Increased in Double Knockout Females?

Though the embryo-sparing effects of 2 structurally distinct aromatase inhibitors and an estrogen receptor blocker/degrader implicate E2 in pregnancy failure in dKOs, we could not show a genotype difference in circulating E2 levels, even using a very sensitive (∼1 pg/mL) mass spectrometry assay. This paradox might derive from the small sample size, the lack of normal distribution in the data, and/or the high proportion of samples with undetectable E1 and/or E2 values, particularly for the control animals (Fig. 6F and 6G). Given the higher median serum E1 and E2 values at 10.5 dpc from dKO vs control animals and the greater E2 content in dKO ovaries, our data are consistent with higher embryonic E2 exposure during the dKO pregnancies. Barely detectable E2 levels, even in pregnant animals, are consistent with the lack of circulating sex hormone–binding globulin in mice (35, 36); therefore, studies that report consistently higher values using immunoassays should be interpreted with caution.

Though we predict that increased ovarian E2 would translate into higher circulating E2 (that we failed to detect), we cannot rule out roles for local E2 synthesis in the placenta or uterus, even though the corpus luteum is the main site of E2 production during rodent gestation (29). In this respect, it is important to note that the aromatase inhibitors rescued pregnancy but appeared to minimally affect ovarian E2 levels (see more later). Aromatase is transiently expressed in the murine uterus at 5.5 and 6.5 dpc, and local estrogen synthesis is necessary for proper decidualization (49). Though not measured directly, we did not see overt evidence of a decidualization defect in dKOs. While placental E2 production later in pregnancy should be considered, aromatase expression in the mid-gestation murine placenta is low to undetectable (29, 49, 50).

The lack of effect of aromatase inhibitors on ovarian E2 content might be apparent more than real. The lowest ovarian E2 levels detected were in 2 of 3 anastrozole-treated females. Given the low sample size in this experiment, it may have been insufficiently powered to detect a significant difference. In contrast, 2 of 3 formestane-treated animals had the highest E2 levels measured in the assay. To our knowledge, the E2 assay used does not cross-react with formestane, making these results more difficult to understand at face value. That said, it is important to note we could not measure E2 levels before and after treatment in the same ovaries and therefore could not directly determine drug efficacy in individual animals. This is an important caveat as there is marked interanimal variation in the number of corpora lutea among dKOs (14). It is possible that corpora lutea number (and therefore E2 levels) may have been, on average, higher among those animals randomly assigned to formestane treatment. Ovarian E2 levels may have been sufficiently reduced by formestane in these animals at 12.5 dpc to increase embryo survival. Indeed, one of the formestane-treated females had 24 live embryos at 12.5 dpc, which suggests that it likely had a large number of corpora lutea and correspondingly high ovarian E2 prior to treatment. After treatment, this female had the lowest ovarian E2 of the formestane-treatment group. Perhaps with larger sample sizes and a direct assessment of corpora lutea number in the treated animals, we could more effectively determine or estimate the efficacy of the aromatase inhibitors on ovarian E2 content on an individual basis. We consider it unlikely that the aromatase inhibitors rescue pregnancy because of off-target effects. Anastrozole and formestane have different molecular structures and their effects converge with those of the estrogen receptor degrader. Therefore, all signs point to increased E2 action in pregnancy loss in dKO mice.

### How Does Estradiol Cause Pregnancy Failure?

Despite considerable effort, we were unable to determine a precise mechanism (or mechanisms) through which excess E2 leads to fetal death. Embryos died at variable time points in dKOs, on or after 10.5 dpc, making it difficult to pinpoint a singular dysregulated process that caused lethality. Most placentae in dKO pregnancies appeared normal at 9.5 dpc, exhibiting standard hallmarks such as chorioallantoic fusion and labyrinthine vasculogenesis (51). Placentae were, nevertheless, notably larger in dKOs. Enlarged placentae can indicate placental inefficiency but are not themselves markers of embryo lethality (52, 53). Indeed, several embryos appeared to develop normally in dKO females at 10.5 dpc.

Excess E2 during mid-gestation (8.5-13.5 dpc) is associated with pregnancy failure in rodents (21, 54), but, to our knowledge, a mechanism has not been delineated. Exogenous E2 given at 10.5 dpc dose-dependently causes fetal death in pregnant wild-type mice (21). Females of some other KO strains with elevated serum estrogens (measured by radioimmunoassay) also exhibit embryo death starting at mid-gestation. For example, 5α-reductase type 1 (*Srd5a1*) KO females lose approximately half their embryos at 10.5 dpc (20). These mice are unable to convert testosterone to dihydrotestosterone and hence produce excess estrogens. As we observed in dKOs, embryo survival is enhanced by aromatase inhibitor or estrogen receptor blocker treatment in *Srd5a1* KOs. Estrogen sulfotransferase (*Sult1e1*)-deficient females also exhibit fetal loss around 12.5 dpc. These mice are impaired in their ability to inactivate estrogens and therefore have elevated serum E2 during mid- to late gestation. Embryo survival can be improved by fulvestrant or low-molecular-weight heparin (LMWH) treatment, suggesting that excess E2 during pregnancy can perturb platelet aggregation. *Sult1e1^−/−^* platelets are more sensitive to agonist-induced activation ex vivo, perhaps contributing to placental thrombosis and fetal death in *Sult1e1* KO females (21). However, in preliminary analyses, LMWH did not increase embryo survival in dKO females in the present study, suggesting that placental thrombosis is likely not responsible for embryo death in our model.

Based on the timeline of most fetal deaths in dKO females (∼10.5 dpc), which coincides with the timing of spiral artery remodeling, we asked whether E2 interferes with placentation—specifically, the trophoblast-mediated remodeling and dilation of the spiral arteries that must occur to accommodate increased blood flow (39, 55, 56). Insufficient E2, through dysregulation of angiogenesis (57-60) and trophoblast expansion (44, 61, 62), has been linked to placentation disorders such as intrauterine growth restriction and preeclampsia in humans (42, 63-65). Conversely, E2 treatment in pregnant rats appears to cause subfertility by reducing spiral artery remodeling, although the underlying mechanism is not well defined (54).

The most mechanistically well-defined model in which excess E2 impairs placentation is in baboons. Maternal serum E2 was (experimentally) prematurely elevated during the first trimester of baboon pregnancy, which resulted in decreased VEGF, increased sFLT1, and suppression of trophoblast invasion into spiral arteries (66-69). These changes culminated in increased maternal blood pressure during the third trimester and decreased uterine and umbilical blood flow after vasochallenge, mirroring human preeclampsia (67). However, several key features of this primate model do not match what we observed in dKO mice. Trophoblast invasion into maternal spiral arteries appears comparable between placentae from controls and dKOs. Placentae from dKO females exhibit increased expression both of proangiogenic and antiangiogenic markers, and any changes in placental vascular density (as assessed by vimentin immunostaining) appear to be modest. Most important, premature elevation of E2 in primates did not lead to fetal death (67). Similarly, mouse models of impaired maternal arterial remodeling or reduced fetoplacental vascular perfusion do not exhibit complete infertility (70-72). Ultimately, it is not clear (1) whether there are any meaningful changes in placental vascularization in dKO females and (2) if decreased vascularization could be the main driver of fully penetrant embryo loss in this model.

### Anti-Inhibin Manipulations to Augment Female Fertility

Attenuating inhibin action in females can increase their fertility. Gonadotrope-specific betaglycan KO mice (13), *Tgfbr3l* global KOs (14), and mice heterozygous for the inhibin-inactivating *Inha^R233A^* mutation (16) all display increased litter sizes. More eggs can be collected from mice superovulated with anti-inhibin serum than mice superovulated with the current gold standard, equine chorionic gonadotropin (73, 74). The percentage of eggs that form blastocysts post fertilization is generally comparable with the two approaches (73, 74). However, attenuating inhibin action can have deleterious effects on the maternal environment of that cycle. When rats are treated with anti-inhibin serum and natural pregnancy is allowed to continue, they deliver more pups than rats treated with normal serum, but two-thirds of the pups are small and either stillborn or die soon after birth (75). Females homozygous for the *Inha^R233A^* mutation also display severe subfertility, with few pups surviving until weaning (16). Interestingly, these females do not exhibit complete infertility, as in the inhibin coreceptor dKO females. Different anti-inhibin manipulations may elevate FSH and therefore circulating E2 to varying degrees, thus explaining the differing phenotypes.

Collectively, these data indicate that anti-inhibin manipulations, used in moderation, are beneficial for female fertility, but a greater attenuation of inhibin activity in gonadotropes may be “too much of a good thing,” leading to fetal death.

## Data Availability

All data are available in the manuscript.

## Abbreviations

dKO: double knockout
dpc: days post coitum
E: embryonic day
E1: estrone
E2: estradiol
ELISA: enzyme-linked immunosorbent assay
FSH: follicle-stimulating hormone
GnRH: gonadotropin-releasing hormone
KO: knockout
LH: luteinizing hormone
LMWH: low-molecular-weight heparin
mRNA: messenger RNA
NBF: neutral buffered formalin
P4: progesterone
PBS: phosphate-buffered saline
TGFβ: transforming growth factor β
VEGF: vascular endothelial growth factor

## References

[1] Edson MA, Nagaraja AK, Matzuk MM. The mammalian ovary from genesis to revelation. Endocr Rev. 2009;30(6):624‐712. 19776209 10.1210/er.2009-0012 PMC2761115

[2] Chun SY, Eisenhauer KM, Minami S, Billig H, Perlas E, Hsueh AJ. Hormonal regulation of apoptosis in early antral follicles: follicle-stimulating hormone as a major survival factor. Endocrinology. 1996;137(4):1447‐1456. 8625923 10.1210/endo.137.4.8625923

[3] Zhou XL, Teng Y, Cao R, et al Rescue from dominant follicle atresia by follicle-stimulating hormone in mice. Genet Mol Res. 2013;12(3):2945‐2952. 24065650 10.4238/2013.August.12.10

[4] Williams CJ, Erickson GF. Morphology and physiology of the ovary. [Updated January 30, 2012]. In: Feingold KR, Ahmed SF, Anawalt B, et al, eds. Endotext [Internet]. MDText.com, Inc.; 2000-. https://www.ncbi.nlm.nih.gov/books/NBK278951/

[5] Gougeon A . Dynamics of follicular growth in the human: a model from preliminary results. Hum Reprod. 1986;1(2):81‐87. 3558758 10.1093/oxfordjournals.humrep.a136365

[6] Zheng W, Zhang H, Liu K. The two classes of primordial follicles in the mouse ovary: their development, physiological functions and implications for future research. Mol Hum Reprod. 2014;20(4):286‐292. 24448914 10.1093/molehr/gau007

[7] Chen Y, Liu Q, Liu R, et al A prepubertal mice model to study the growth pattern of early ovarian follicles. Int J Mol Sci. 2021;22(10):5130. 34066233 10.3390/ijms22105130 PMC8151218

[8] Lunenfeld B, Bilger W, Longobardi S, Alam V, D'Hooghe T, Sunkara SK. The development of gonadotropins for clinical use in the treatment of infertility. Front Endocrinol (Lausanne). 2019;10:429. 31333582 10.3389/fendo.2019.00429 PMC6616070

[9] Ongaro L, Zhou X, Wang Y, et al Muscle-derived myostatin is a major endocrine driver of follicle-stimulating hormone synthesis. Science. 2025;387(6731):329‐336. 39818879 10.1126/science.adi4736 PMC12199281

[10] Bernard DJ, Schang G, Li Y, Ongaro L, Thompson TB. Activins and inhibins in female reproduction. In: Skinner MK, ed. Encyclopedia of Reproduction. 2nd ed. Academic Press; 2018:202‐210.

[11] Ling N, Ying SY, Ueno N, et al Pituitary FSH is released by a heterodimer of the beta-subunits from the two forms of inhibin. Nature. 1986;321(6072):779‐782. 3086749 10.1038/321779a0

[12] Lewis KA, Gray PC, Blount AL, et al Betaglycan binds inhibin and can mediate functional antagonism of activin signalling. Nature. 2000;404(6776):411‐414. 10746731 10.1038/35006129

[13] Li Y, Fortin J, Ongaro L, et al Betaglycan (TGFBR3) functions as an inhibin A, but not inhibin B, coreceptor in pituitary gonadotrope cells in mice. Endocrinology. 2018;159(12):4077‐4091. 30364975 10.1210/en.2018-00770 PMC6372943

[14] Brule E, Wang Y, Li Y, et al TGFBR3L is an inhibin B co-receptor that regulates female fertility. Sci Adv. 2021;7(51):eabl4391. 34910520 10.1126/sciadv.abl4391 PMC8673766

[15] Schang G, Ongaro L, Schultz H, et al Murine FSH production Depends on the activin type II receptors ACVR2A and ACVR2B. Endocrinology. 2020;161(7):bqaa056. 32270195 10.1210/endocr/bqaa056 PMC7286621

[16] Walton KL, Goney MP, Peppas Z, et al Inhibin inactivation in female mice leads to elevated FSH levels, ovarian overstimulation, and pregnancy loss. Endocrinology. 2022;163(4):bqac025. 35255139 10.1210/endocr/bqac025 PMC9272799

[17] Wen S, Schwarz JR, Niculescu D, et al Functional characterization of genetically labeled gonadotropes. Endocrinology. 2008;149(6):2701‐2711. 18325995 10.1210/en.2007-1502

[18] Lin YF, Brule E, Ongaro L, et al 2025. Data from: Data from: Loss of inhibin negative feedback to pituitary gonadotropes leads to enhanced ovulation but pregnancy failure in mice. Figshare Digital Repository. doi: 10.6084/m9.figshare.29206229. PMC12481700 40988449

[19] Theiler K . The House Mouse: Atlas of Embryonic Development. Springer; 1989.

[20] Mahendroo MS, Cala KM, Landrum DP, Russell DW. Fetal death in mice lacking 5alpha-reductase type 1 caused by estrogen excess. Mol Endocrinol. 1997;11(7):917‐927. 9178751 10.1210/mend.11.7.9933

[21] Tong MH, Jiang H, Liu P, Lawson JA, Brass LF, Song WC. Spontaneous fetal loss caused by placental thrombosis in estrogen sulfotransferase-deficient mice. Nat Med. 2005;11(2):153‐159. 15685171 10.1038/nm1184

[22] Schindelin J, Arganda-Carreras I, Frise E, et al Fiji: an open-source platform for biological-image analysis. Nat Methods. 2012;9(7):676‐682. 22743772 10.1038/nmeth.2019 PMC3855844

[23] Steyn FJ, Wan Y, Clarkson J, Veldhuis JD, Herbison AE, Chen C. Development of a methodology for and assessment of pulsatile luteinizing hormone secretion in juvenile and adult male mice. Endocrinology. 2013;154(12):4939‐4945. 24092638 10.1210/en.2013-1502 PMC5398599

[24] Ongaro L, Alonso CAI, Zhou X, et al Development of a highly sensitive ELISA for measurement of FSH in serum, plasma, and whole blood in mice. Endocrinology. 2021;162(4):bqab014. 33475143 10.1210/endocr/bqab014 PMC7894055

[25] Cinar O, Demir B, Dilbaz S, Saltek S, Aydin S, Goktolga U. Does empty zona pellucida indicate poor ovarian response on intra cytoplasmic sperm injection cycles? Gynecol Endocrinol. 2012;28(5):341‐344. 22132865 10.3109/09513590.2011.631632

[26] Rienzi L, Balaban B, Ebner T, Mandelbaum J. The oocyte. Hum Reprod. 2012;27(suppl 1):i2‐i21. 22811312 10.1093/humrep/des200

[27] Atzmon Y, Michaeli M, Poltov D, et al Degenerated oocyte in the cohort adversely affects IVF outcome. J Ovarian Res. 2020;13(1):109. 32943105 10.1186/s13048-020-00708-6 PMC7495854

[28] Galosy SS, Talamantes F. Luteotropic actions of placental lactogens at midpregnancy in the mouse. Endocrinology. 1995;136(9):3993‐4003. 7649108 10.1210/endo.136.9.7649108

[29] Malassine A, Frendo JL, Evain-Brion D. A comparison of placental development and endocrine functions between the human and mouse model. Hum Reprod Update. 2003;9(6):531‐539. 14714590 10.1093/humupd/dmg043

[30] Wetendorf M, Wu SP, Wang X, et al Decreased epithelial progesterone receptor A at the window of receptivity is required for preparation of the endometrium for embryo attachment. Biol Reprod. 2017;96(2):313‐326. 28203817 10.1095/biolreprod.116.144410 PMC6225975

[31] Lee KY, Jeong JW, Wang J, et al Bmp2 is critical for the murine uterine decidual response. Mol Cell Biol. 2007;27(15):5468‐5478. 17515606 10.1128/MCB.00342-07 PMC1952078

[32] Lee K, Jeong J, Kwak I, et al Indian hedgehog is a major mediator of progesterone signaling in the mouse uterus. Nat Genet. 2006;38(10):1204‐1209. 16951680 10.1038/ng1874

[33] Moggs JG, Tinwell H, Spurway T, et al Phenotypic anchoring of gene expression changes during estrogen-induced uterine growth. Environ Health Perspect. 2004;112(9):1589‐1606. 15598610 10.1289/txg.7345 PMC1247656

[34] Ratajczak CK, Boehle KL, Muglia LJ. Impaired steroidogenesis and implantation failure in Bmal1-/- mice. Endocrinology. 2009;150(4):1879‐1885. 19056819 10.1210/en.2008-1021 PMC5393263

[35] Janne M, Deol HK, Power SG, Yee SP, Hammond GL. Human sex hormone-binding globulin gene expression in transgenic mice. Mol Endocrinol. 1998;12(1):123‐136. 9440816 10.1210/mend.12.1.0050

[36] Laurent MR, Hammond GL, Blokland M, et al Sex hormone-binding globulin regulation of androgen bioactivity in vivo: validation of the free hormone hypothesis. Sci Rep. 2016;6(1):35539. 27748448 10.1038/srep35539 PMC5066276

[37] Haisenleder DJ, Schoenfelder AH, Marcinko ES, Geddis LM, Marshall JC. Estimation of estradiol in mouse serum samples: evaluation of commercial estradiol immunoassays. Endocrinology. 2011;152(11):4443‐4447. 21933867 10.1210/en.2011-1501 PMC3198998

[38] Mahendroo MS, Porter A, Russell DW, Word RA. The parturition defect in steroid 5alpha-reductase type 1 knockout mice is due to impaired cervical ripening. Mol Endocrinol. 1999;13(6):981‐992. 10379896 10.1210/mend.13.6.0307

[39] Cross JC, Hemberger M, Lu Y, et al Trophoblast functions, angiogenesis and remodeling of the maternal vasculature in the placenta. Mol Cell Endocrinol. 2002;187(1-2):207‐212. 11988329 10.1016/s0303-7207(01)00703-1

[40] Cross J . Mechanisms of trophoblast differentiationand maternal–fetal interactions in the mouse. In: Aplin JD, Fazleabas AT, Glasser SR, Giudice LC, eds. The Endometrium. 2nd ed. CRC Press; 2008:466‐475.

[41] Albrecht ED, Robb VA, Pepe GJ. Regulation of placental vascular endothelial growth/permeability factor expression and angiogenesis by estrogen during early baboon pregnancy. J Clin Endocrinol Metab. 2004;89(11):5803‐5809. 15531545 10.1210/jc.2004-0479

[42] Berkane N, Liere P, Oudinet JP, et al From pregnancy to preeclampsia: a key role for estrogens. Endocr Rev. 2017;38(2):123‐144. 28323944 10.1210/er.2016-1065

[43] Mandala M . Influence of estrogens on uterine vascular adaptation in normal and preeclamptic pregnancies. Int J Mol Sci. 2020;21(7):2592. 32276444 10.3390/ijms21072592 PMC7177259

[44] Rusidze M, Faure MC, Sicard P, et al Loss of function of the maternal membrane oestrogen receptor ERalpha alters expansion of trophoblast cells and impacts mouse fertility. Development. 2022;149(19):dev200683. 36239412 10.1242/dev.200683 PMC9720743

[45] Simmons DG, Fortier AL, Cross JC. Diverse subtypes and developmental origins of trophoblast giant cells in the mouse placenta. Dev Biol. 2007;304(2):567‐578. 17289015 10.1016/j.ydbio.2007.01.009

[46] Simmons DG . 12—Postimplantation Development of the chorioallantoic placenta. In: Croy BA, Yamada AT, DeMayo FJ, Adamson SL, eds. The Guide to Investigation of Mouse Pregnancy. Academic Press; 2014:143‐161.

[47] He Y, Smith SK, Day KA, Clark DE, Licence DR, Charnock-Jones DS. Alternative splicing of vascular endothelial growth factor (VEGF)-R1 (FLT-1) pre-mRNA is important for the regulation of VEGF activity. Mol Endocrinol. 1999;13(4):537‐545. 10194760 10.1210/mend.13.4.0265

[48] Maynard SE, Min JY, Merchan J, et al Excess placental soluble fms-like tyrosine kinase 1 (sFlt1) may contribute to endothelial dysfunction, hypertension, and proteinuria in preeclampsia. J Clin Invest. 2003;111(5):649‐658. 12618519 10.1172/JCI17189 PMC151901

[49] Das A, Mantena SR, Kannan A, Evans DB, Bagchi MK, Bagchi IC. De novo synthesis of estrogen in pregnant uterus is critical for stromal decidualization and angiogenesis. Proc Natl Acad Sci U S A. 2009;106(30):12542‐12547. 19620711 10.1073/pnas.0901647106 PMC2718343

[50] Marsh B, Blelloch R. Single nuclei RNA-seq of mouse placental labyrinth development. Elife. 2020;9:e60266. 33141023 10.7554/eLife.60266 PMC7669270

[51] Bolon B . 14—Pathology Analysis of the placenta. In: Croy BA, Yamada AT, DeMayo FJ, Adamson SL, eds. The Guide to Investigation of Mouse Pregnancy. Academic Press; 2014:175‐188.

[52] Coan PM, Angiolini E, Sandovici I, Burton GJ, Constancia M, Fowden AL. Adaptations in placental nutrient transfer capacity to meet fetal growth demands depend on placental size in mice. J Physiol. 2008;586(18):4567‐4576. 18653658 10.1113/jphysiol.2008.156133 PMC2614013

[53] Fowden AL, Sferruzzi-Perri AN, Coan PM, Constancia M, Burton GJ. Placental efficiency and adaptation: endocrine regulation. J Physiol. 2009;587(14):3459‐3472. 19451204 10.1113/jphysiol.2009.173013 PMC2742275

[54] Furukawa S, Hayashi S, Usuda K, et al Effect of estrogen on rat placental development depending on gestation stage. Exp Toxicol Pathol. 2013;65(5):695‐702. 23164498 10.1016/j.etp.2012.09.002

[55] Adamson SL, Lu Y, Whiteley KJ, et al Interactions between trophoblast cells and the maternal and fetal circulation in the mouse placenta. Dev Biol. 2002;250(2):358‐373. 12376109 10.1016/s0012-1606(02)90773-6

[56] Hemberger M, Nozaki T, Masutani M, Cross JC. Differential expression of angiogenic and vasodilatory factors by invasive trophoblast giant cells depending on depth of invasion. Dev Dyn. 2003;227(2):185‐191. 12761846 10.1002/dvdy.10291

[57] Lam C, Lim KH, Karumanchi SA. Circulating angiogenic factors in the pathogenesis and prediction of preeclampsia. Hypertension. 2005;46(5):1077‐1085. 16230516 10.1161/01.HYP.0000187899.34379.b0

[58] Herve MA, Meduri G, Petit FG, et al Regulation of the vascular endothelial growth factor (VEGF) receptor Flk-1/KDR by estradiol through VEGF in uterus. J Endocrinol. 2006;188(1):91‐99. 16394178 10.1677/joe.1.06184

[59] Kim M, Park HJ, Seol JW, et al VEGF-A regulated by progesterone governs uterine angiogenesis and vascular remodelling during pregnancy. EMBO Mol Med. 2013;5(9):1415‐1430. 23853117 10.1002/emmm.201302618 PMC3799495

[60] Fan X, Rai A, Kambham N, et al Endometrial VEGF induces placental sFLT1 and leads to pregnancy complications. J Clin Invest. 2014;124(11):4941‐4952. 25329693 10.1172/JCI76864 PMC4347223

[61] He WH, Jin MM, Liu AP, et al Estradiol promotes trophoblast viability and invasion by activating SGK1. Biomed Pharmacother. 2019;117:109092. 31203134 10.1016/j.biopha.2019.109092

[62] Wang H, Bocca S, Anderson S, et al Sex steroids regulate epithelial-stromal cell cross talk and trophoblast attachment invasion in a three-dimensional human endometrial culture system. Tissue Eng Part C Methods. 2013;19(9):676‐687. 23320930 10.1089/ten.TEC.2012.0616

[63] Bukovsky A, Cekanova M, Caudle MR, et al Expression and localization of estrogen receptor-alpha protein in normal and abnormal term placentae and stimulation of trophoblast differentiation by estradiol. Reprod Biol Endocrinol. 2003;1(1):13. 12646062 10.1186/1477-7827-1-13 PMC151787

[64] Shimodaira M, Nakayama T, Sato I, et al Estrogen synthesis genes CYP19A1, HSD3B1, and HSD3B2 in hypertensive disorders of pregnancy. Endocrine. 2012;42(3):700‐707. 22638611 10.1007/s12020-012-9699-7

[65] Acikgoz S, Bayar UO, Can M, et al Levels of oxidized LDL, estrogens, and progesterone in placenta tissues and serum paraoxonase activity in preeclampsia. Mediators Inflamm. 2013;2013:862982. 23606795 10.1155/2013/862982 PMC3625559

[66] Bonagura TW, Pepe GJ, Enders AC, Albrecht ED. Suppression of extravillous trophoblast vascular endothelial growth factor expression and uterine spiral artery invasion by estrogen during early baboon pregnancy. Endocrinology. 2008;149(10):5078‐5087. 18566115 10.1210/en.2008-0116 PMC2582926

[67] Aberdeen GW, Bonagura TW, Harman CR, Pepe GJ, Albrecht ED. Suppression of trophoblast uterine spiral artery remodeling by estrogen during baboon pregnancy: impact on uterine and fetal blood flow dynamics. Am J Physiol Heart Circ Physiol. 2012;302(10):H1936‐H1944. 22427518 10.1152/ajpheart.00590.2011 PMC3362108

[68] Babischkin JS, Aberdeen GW, Lindner JR, Bonagura TW, Pepe GJ, Albrecht ED. Vascular endothelial growth factor delivery to placental basal plate promotes uterine artery remodeling in the primate. Endocrinology. 2019;160(6):1492‐1505. 31002314 10.1210/en.2019-00059 PMC6542484

[69] Aberdeen GW, Babischkin JS, Lindner JR, Pepe GJ, Albrecht ED. Placental sFlt-1 gene delivery in early primate pregnancy suppresses uterine spiral artery remodeling. Endocrinology. 2022;163(4):bqac012. 35134145 10.1210/endocr/bqac012 PMC8896163

[70] Guimond MJ, Luross JA, Wang B, Terhorst C, Danial S, Croy BA. Absence of natural killer cells during murine pregnancy is associated with reproductive compromise in TgE26 mice. Biol Reprod. 1997;56(1):169‐179. 9002646 10.1095/biolreprod56.1.169

[71] van der Heijden OW, Essers YP, Fazzi G, Peeters LL, De Mey JG, van Eys GJ. Uterine artery remodeling and reproductive performance are impaired in endothelial nitric oxide synthase-deficient mice. Biol Reprod. 2005;72(5):1161‐1168. 15659709 10.1095/biolreprod.104.033985

[72] Lacko LA, Hurtado R, Hinds S, Poulos MG, Butler JM, Stuhlmann H. Altered feto-placental vascularization, feto-placental malperfusion and fetal growth restriction in mice with Egfl7 loss of function. Development. 2017;144(13):2469‐2479. 28526753 10.1242/dev.147025 PMC5536866

[73] Wuri L, Agca C, Agca Y. Morphometric, subcellular, in vitro fertilisation and embryonic developmental assessment of mouse oocytes produced by anti-inhibin serum or pregnant mare serum gonadotrophin superovulation. Reprod Fertil Dev. 2020;32(5):474‐483. 31972126 10.1071/RD19131 PMC8020730

[74] Hasegawa A, Mochida K, Inoue H, et al High-yield superovulation in adult mice by anti-inhibin serum treatment combined with estrous cycle synchronization. Biol Reprod. 2016;94(1):21. 26632610 10.1095/biolreprod.115.134023

[75] Rivier C, Vale W. Immunoneutralization of endogenous inhibin modifies hormone secretion and ovulation rate in the rat. Endocrinology. 1989;125(1):152‐157. 2500324 10.1210/endo-125-1-152

